# Genetical modification on adipose-derived stem cells facilitates facial nerve regeneration

**DOI:** 10.18632/aging.101790

**Published:** 2019-02-06

**Authors:** Jian Tan, Yipin Xu, Fang Han, Xinhai Ye

**Affiliations:** 1 Department of Plastic Surgery, Shanghai Tenth People's Hospital, Tongji University School of Medicine, Shanghai 200072, China; 2 Department of Facial Plastic and Reconstructive Surgery, Eye and ENT Hospital of Fudan University, Shanghai 200031, China

**Keywords:** facial nerve regeneration, adipose-derived stem cells (ASCs), PLOD1, miR-449

## Abstract

Adipose-derived stem cells (ASCs) have a demonstrative therapeutic potential in aging-associated facial nerve regeneration, in which ASCs work as a source of Schwann cells therapy as an alternative to autologous nerve grafts. However, the transplantation of ASCs may induce local fibrosis, which causes inferior outcome. Here, we aimed to use genetic modification approaches to reduce the fibrogenic properties of ASCs to improve their therapeutic effects on facial nerve regeneration. Since procollagen-lysine 1, 2-oxoglutarate 5-dioxygenase 1 (PLOD1) is essential for hydroxylation of lysine residues in collagen telopeptides and for collagen pyridinoline cross-link formation during fibrosis, and since we found that ASCs expressed high levels of PLOD1, we depleted PLOD1 in ASCs by expression of either a short-hair interfering RNA for PLOD1 (shPLOD1) or a microRNA-449 (miR-449), the latter of which targets PLOD1 mRNA to suppress protein translation. Transplantation of either ASCs-shPLOD1 or ASCs-miR-449 or ASCs-control to repair a 6mm-gap in rat facial nerve was compared. Either ASCs-shPLOD1 or ASCs-miR-449 exhibited a better facial nerve function. Mechanistically, ASCs-shPLOD1 or ASCs-miR-449 significantly and similarly reduced the fibrosis in the injured region, likely through suppression of reactive oxygen species (ROS) and activation of myofibroblasts.

## Introduction

Patients with aging-associated facial nerve injuries may face life-changing problems that require high cost of social and health care [1]. To fundamentally solve the issue, promotion of facial nerve regeneration is an attractive strategy, which relies predominantly on Schwann cells that possess certain regenerative capability [2]. Traditionally, clinical repairing of a nerve gap needs scarification of a comparable length of nerve from self to develop a bioengineered nerve graft for replacement of the lost one, which often result in poor functional recovery [2].

Recently, alternative bioengineered nerve grafts have been used to guide the regeneration of axons across nerve gaps by the components in the graft, e.g. extracellular matrix, certain growth factors, some pharmaceutical adjuvants and transplanted cells [3]. Schwann cells play pivotal roles in peripheral nerve regeneration, by releasing important growth factors and facilitating in re-myelination [4]. Thus, adipose-derived stem cells (ASCs), which have been shown to have potential of differentiating into Schwann cells *in vitro* and *in vivo*, hold promise to be the most appropriate cells to replace Schwann cells or autologous nerve in facial nerve repair [5]. ASCs have the advantage of good abundancy, accessibility and characteristics [6–8]. Besides differentiation into Schwann cells, ASCs may also function in nerve regeneration sites through production and secretion of trophic factors to enhance regeneration. Moreover, ASCs may adjust local inflammation [9–12]. Interestingly, recent studies have shown that the function of ASCs may be further improved through genetic modification in treating different diseases [13–16].

ASCs can be differentiated towards different lineages, including adipogenic, osteogenic, chondrogenic, myogenic, and neurogenic lineages [17]. This property decides that ASCs may have multiple characteristics that may either be beneficial or detrimental to facial nerve regeneration. Specially, ASCs overall inhibits fibrosis, but they also possess characteristics that are fibrogenic, which may be harmful to the regeneration [18]. Fibrosis can be histologically distinguished from normal tissue by the arrangement of collagen fibers, presence of myofibroblasts that express α-smooth muscle actin (αSMA), and high expression of transforming growth factor β1 (TGFβ1) [19]. Common pathways of fibrosis include signaling through transforming growth factor β (TGFβ), connective tissue growth factor (CTGF), interleukin-4 (IL-4), IL-13, platelet-derived growth factor (PDGF), and osteopontin [20]. Moreover, procollagen-lysine 1, 2-oxoglutarate 5-dioxygenase 1 (PLOD1) is essential for regulation of hydroxylation of lysine residues in collagen telopeptides and for collagen pyridinoline cross-link formation during fibrosis [21]. Fibrosis may cause function impairment, patient discomfort and psychological stress, resulting in decreases in the life quality of the patients [22]. Hence, suppression of fibrogenic effects of ASCs may further improve their therapeutic effects on facial nerve regeneration, which was tested in the current study.

Since we found that ASCs expressed high levels of PLOD1, we depleted PLOD1 in ASCs by expression of either a short-hair interfering RNA for PLOD1 (shPLOD1) or a microRNA-449 (miR-449), the latter of which targets PLOD1 mRNA to suppress protein translation. Transplantation of either ASCs-shPLOD1 or ASCs-miR-449 or control ASCs-scr to repair a 6mm-gap in rat facial nerve was compared. Either ASCs-shPLOD1 or ASCs-miR-449 exhibited a better facial nerve function. Mechanistically, ASCs-shPLOD1 or ASCs-miR-449 significantly and similarly reduced the fibrosis in the injured region, likely through suppression of reactive oxygen species (ROS) and activation of myofibroblasts.

## RESULTS

### MiR-449 is a PLOD1-targetting miRNA low expressed in ASCs

**Figure 1:**
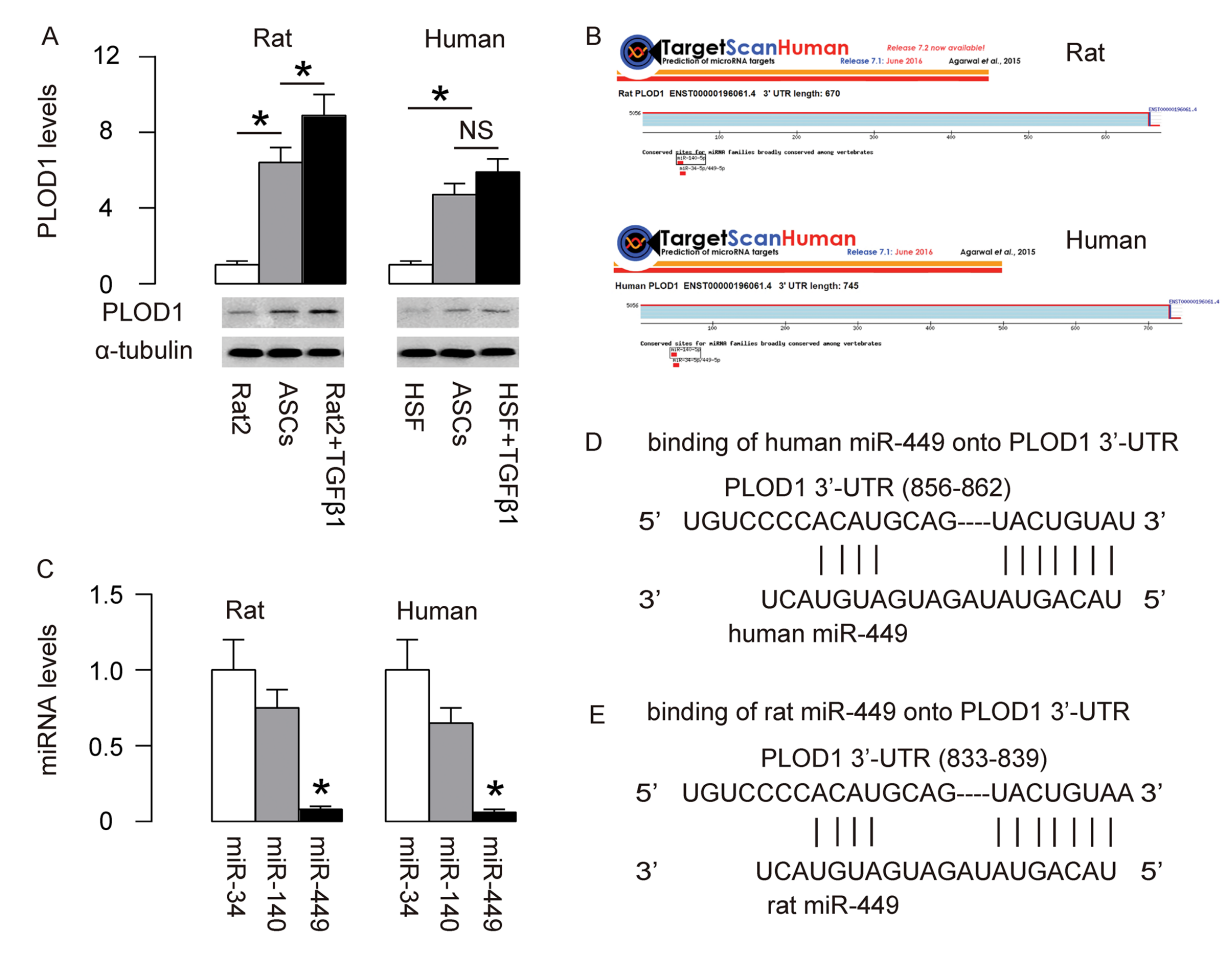
**MiR-449 is a PLOD1-targetting miRNA low expressed in ASCs.** (**A**) PLOD1 protein levels were examined in rat and human ASCs by Western blotting. Rat2 and HSFs cells treated with/without TGFβ1 were used as controls. (**B**) Bioinformatics tools were used to find 3 PLOD1-targeting miRNAs (miR-34, miR-140 and miR-449) conserved in rat and human. (**C**) RT-qPCR for the 3 PLOD1-targeting miRNAs (miR-34, miR-140 and miR-449) in rat and human ASCs. (**D-E**) The binding sites of miR-449 on human (D) and rat (E) PLOD1 3’-UTR were shown. *p<0.05. NS: non-significant. N=5.

PLOD1 is a known pro-fibrotic factor, which plays an essential role in regulation of hydroxylation of lysine residues in collagen telopeptides and for collagen pyridinoline cross-link formation during fibrosis [21]. In order to increase the anti-fibrotic potential of ASCs, we first examined if PLOD1 levels in ASCs may be high and thus could be downregulated through genetic editing of ASCs. PLOD1 in rat and human ASCs were thus compared to Rat2 and HSFs treated with/without TGFβ1, correspondingly. Interestingly, we detected higher levels of PLOD1 in both rat and human ASCs than non-TGFβ1-treated fibroblasts (Figure 1A). Next, we aimed to figure out if the levels of PLOD1 in ASCs may be downregulated by miRNAs. Using bioinformatics tools, we found 3 PLOD1-targeting miRNAs (miR-34, miR-140 and miR-449) conserved in rat and human (Figure 1B). Then we examined the expression levels of these miRNAs in rat and human ASCs, and found that the levels of miR-449 were significantly lower than the other two miRNAs (Figure 1C). Since overexpression of a low-level miRNA should be more effective than any high-level miRNA on suppression of the target gene, we focused on miR-449 in the current study. The binding sites of miR-449 on human and rat PLOD1 3’-UTR were shown (Figure 1D).

### MiR-449 targets 3’-UTR of PLOD1 mRNA to inhibit its protein translation in ASCs

**Figure 2:**
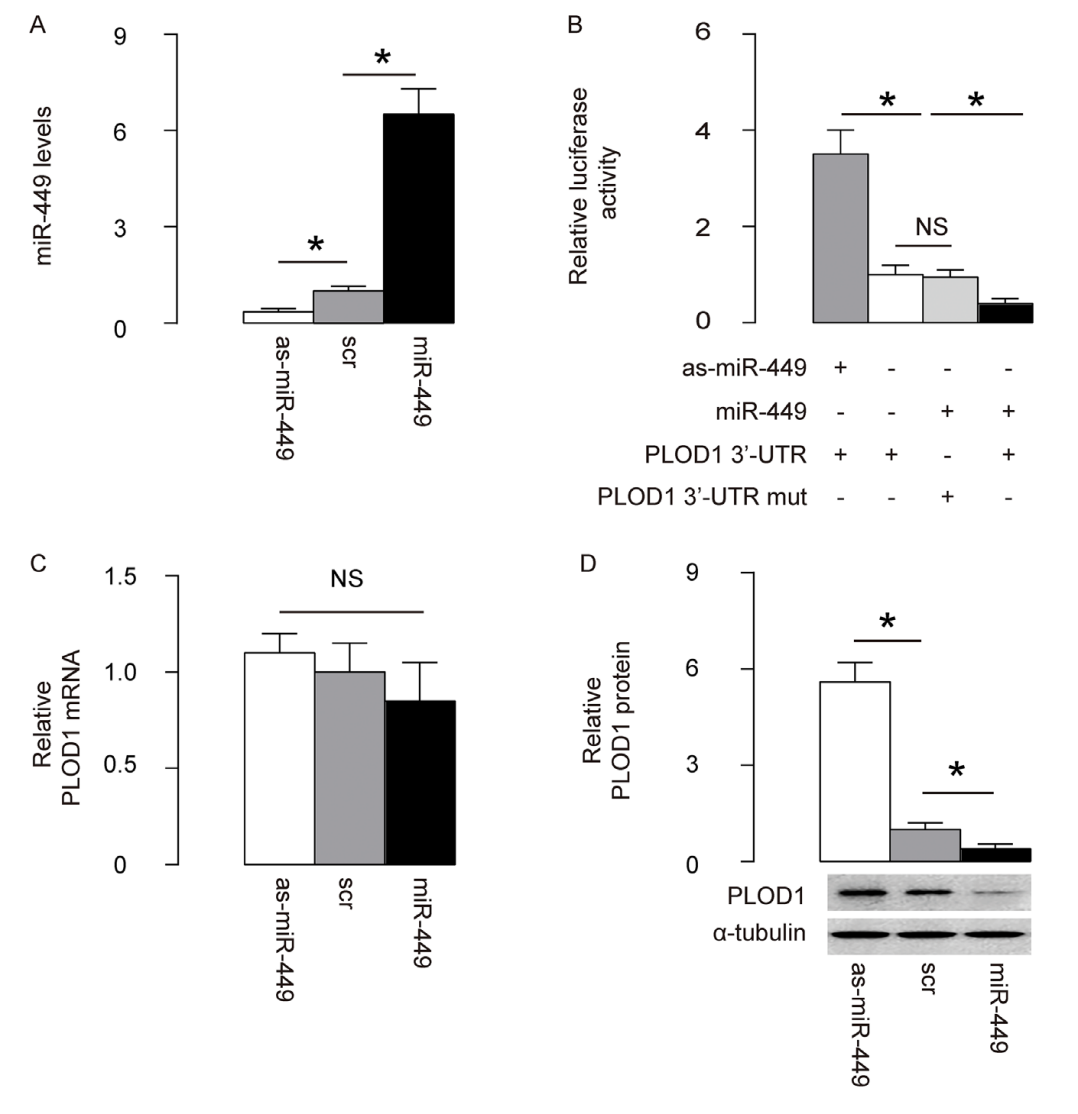
**MiR-449 targets 3’-UTR of PLOD1 mRNA to inhibit its protein translation in ASCs.** (**A**) Rat ASCs cells were transfected with plasmids carrying miR-449 or as-miR-449 or scr as a control. RT-qPCR for miR-449 was done. (**B**) The intact 3'-UTR of wildtype PLOD1 mRNA (wt PLOD1 3'-UTR) and the 3'-UTR of PLOD1 mRNA with a mutant at miR-449-binding site (mut PLOD1 3'-UTR) were respectively cloned into luciferase reporter plasmids. Rat ASCs cells were then co-transfected with one plasmid from miR-449/as-miR-449/null plasmids and one plasmid from either wt PLOD1 3'-UTR or mut PLOD1 3'-UTR, and subsequently subjected to a dual luciferase reporter assay. (**C**) RT-qPCR for PLOD1 mRNA in miR-449-modified rat ASCs. (**D**) Western blotting for PLOD1 protein in miR-449-modified rat ASCs. *p<0.05. NS: non-significant. N=5.

Next, we examined whether the bindings of miR-449 to 3’UTR of PLOD1 mRNA may affect protein translation of PLOD1. First, we transfected rat ASCs cells with plasmids carrying miR-449 or as-miR-449 or scrambled sequence (scr) as a control. RT-qPCR for miR-449 was performed in these transfected cells to confirm the alteration of miR-449 levels (Figure 2A). Then, the intact 3'-UTR of wildtype PLOD1 mRNA (wt PLOD1 3'-UTR) and the 3'-UTR of PLOD1 mRNA with a mutant at miR-449-binding site (mut PLOD1 3'-UTR) were respectively cloned into luciferase reporter plasmids. Rat ASCs cells were then co-transfected with one plasmid from miR-449/as-miR-449/null plasmids and one plasmid from either wt PLOD1 3'-UTR or mut PLOD1 3'-UTR, and subsequently subjected to a dual luciferase reporter assay. We found that depletion of miR-449 increased luciferase activity of wt PLOD1 3'-UTR, while overexpression of miR-449 reduced luciferase activity of wt PLOD1 3'-UTR but had no effects on mut PLOD1 3'-UTR (Figure 2B). Moreover, alteration of miR-449 levels in rat ASCs did not change the mRNA levels of PLOD1 (Figure 2C). However, upregulation of miR-449 significantly decreased PLOD1 protein, while downregulation of miR-449 significantly increased PLOD1 protein in rat ASCs (Figure 2D). These results suggest that miR-449 specifically targets 3’-UTR of PLOD1 mRNA to inhibit its translation in ASCs.

### Preparation of ASCs-miR-449 and ASCs-shPLOD1

**Figure 3:**
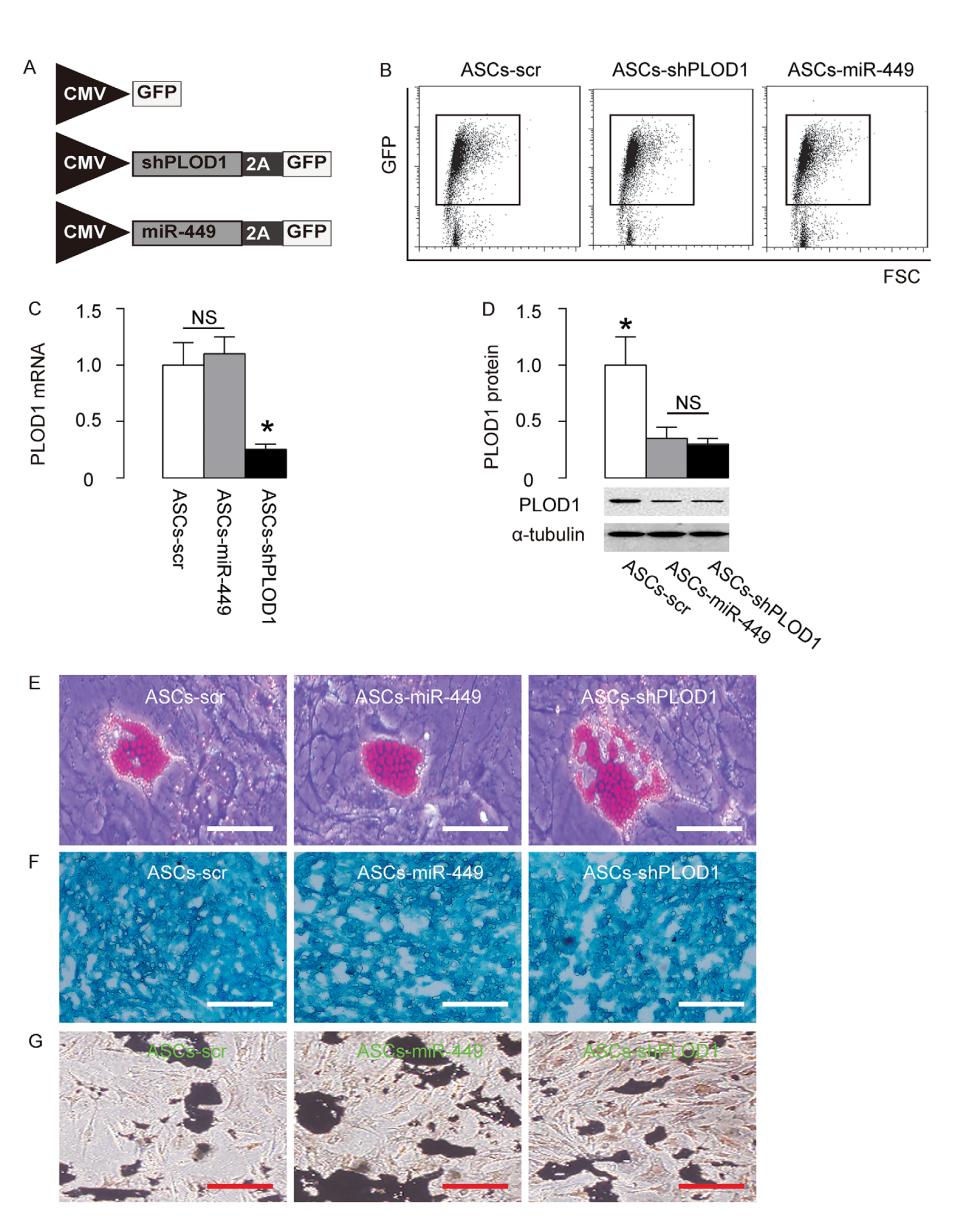
**Preparation of ASCs-miR-449 and ASCs-shPLOD1.** (**A**) Schematic of AAVs. 1. an AAV carrying short-hairpin small interfering RNA for PLOD1 (shPLOD1) and a GFP reporter under control of a CMV promoter. 2. an AAV carrying miR-449 and a GFP reporter under control of a CMV promoter. 3. an AAV carrying GFP and scr. (**B**) Transduced rat ASCs were purified based on GFP expression by flow cytometry. (**C**) RT-qPCR for PLOD1 levels in ASCs-miR-449, ASCs-shPLOD1 and control ASCs-scr. (**D**) Western blotting for PLOD1 levels in ASCs-miR-449, ASCs-shPLOD1 and control ASCs-scr. (**E**) Oil red O staining to evaluate adipogenic induction. (**F**) Alcian blue staining to evaluate chondrogenetic induction. (**G**) Von Kossa staining to evaluate osteogenic induction. *p<0.05. NS: non-significant. N=5. Scale bars are 50µm.

Since we detected high levels of PLOD1 in ASCs, here we aimed to increase the anti-scar potential of ASCs during wound healing through suppression of PLOD1. Two approaches were used. First, ASCs were transduced with an AAV carrying short-hairpin small interfering RNA for PLOD1 (shPLOD1). Second, ASCs were transduced with an AAV carrying miR-449. Both viruses also contained a GFP reporter. ASCs transduced with an AAV carrying GFP and scr were used as a control (Figure 3A). After transduction of rat ASCs were these AAVs, the successfully transduced cells were purified based on GFP expression by flow cytometry (Figure 3B). PLOD1 levels were determined in these modified ASCs by RT-qPCR (Figure 3C) and Western blotting (Figure 3D). We found that expression of miR-449 in ASCs did not alter PLOD1 mRNA (Figure 3C), but significantly decreased PLOD1 protein (Figure 3D). On the other hand, suppression of PLOD1 in ASCs by shRNA significantly decreased both PLOD1 mRNA (Figure 3C) and PLOD1 protein (Figure 3D). Moreover, the PLOD1 protein in ASCs by miR-449 and by shPLOD1 was no difference (Figure 3D). Hence, two approaches may similarly alter PLOD1 protein levels. Finally, the ASCs-miR-449, ASCs-shPLOD1 and control ASCs-scr were subjected to differentiation assays to confirm the MSC-phenotype after genetic editing. We performed Oil red O staining to evaluate adipogenic induction (Figure 3E), Alcian blue staining to evaluate chondrogenetic induction (Figure 3F) and Von Kossa staining to evaluate osteogenic induction (Figure 3G). Our data confirmed the maintenance of the MSC phenotype of ASCs-miR-449, ASCs-shPLOD1 and control ASCs-scr.

### Transplantation of either ASCs-miR-449 or ASCs-shPLOD1 exerts better functional facial nerve regeneration than ASCs in rats

**Figure 4:**
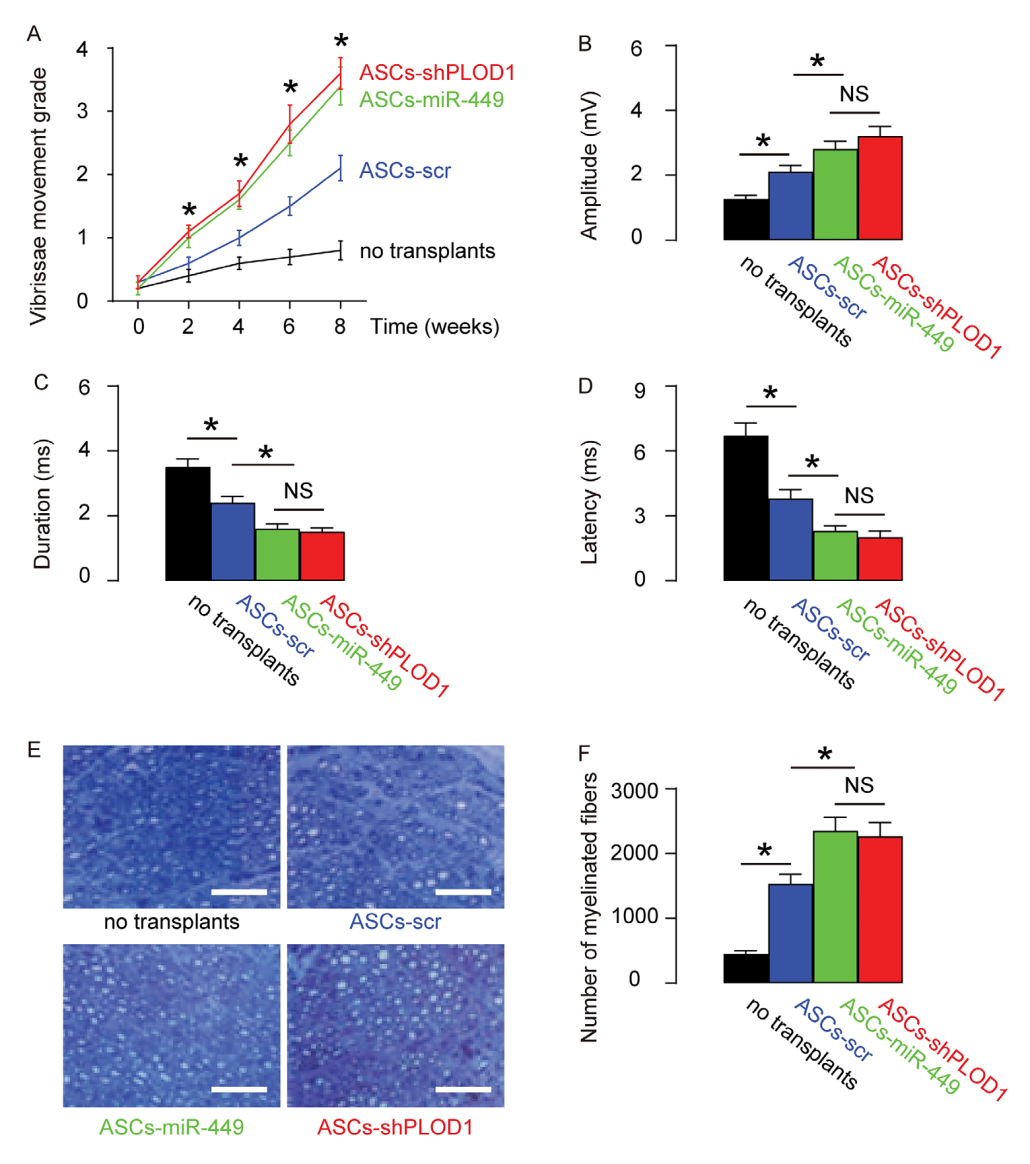
**Transplantation of either ASCs-miR-449 or ASCs-shPLOD1 exerts better functional facial nerve regeneration than ASCs in rats.** Transplantation of either ASCs-shPLOD1 or ASCs-miR-449 or ASCs-control to repair a 6mm-gap in rat facial nerve was compared. The rats were kept for 8 weeks after surgery. (**A**) The measurement of vibrissae movement grade in 8 weeks after surgery. (**B-D**) CAMPs analysis at 8 weeks, showing amplitude (**B**), duration (**C**) and latency (**D**) values. (**E-F**) The number of the myelinated fibers, shown by representative images (**E**), and by quantification (**F**). *p<0.05. NS: non-significant. N=10. Scale bars are 50µm.

Next, we assessed if ASCs-miR-449 or ASCs-shPLOD1 may have therapeutic effects on facial nerve regeneration than ASCs. Thus, transplantation of either ASCs-shPLOD1 or ASCs-miR-449 or ASCs-control to repair a 6mm-gap in rat facial nerve was compared. The rats were kept for 8 weeks after surgery. The measurement of vibrissae movement grade showed that either ASCs-shPLOD1 or ASCs-miR-449 exhibited a better facial nerve function than ASCs-scr (Figure 4A). CAMPs analysis at 8 weeks showed that either ASCs-shPLOD1 or ASCs-miR-449 exhibited a greater amplitude (Figure 4B), a shorter duration (Figure 4C) and a shorter latency (Figure 4D), than ASCs-scr. Moreover, either ASCs-shPLOD1 or ASCs-miR-449 significantly increased the myelinated fibers than ASCs-scr, shown by representative images (Figure 4E), and by quantification (Figure 4F). Together, these data suggest that transplantation of either ASCs-miR-449 or ASCs-shPLOD1 exerts better functional facial nerve regeneration than ASCs in rats.

### Transplantation of either ASCs-miR-449 or ASCs-shPLOD1 reduces levels of fibrosis than ASCs during facial nerve regeneration in rats

**Figure 5:**
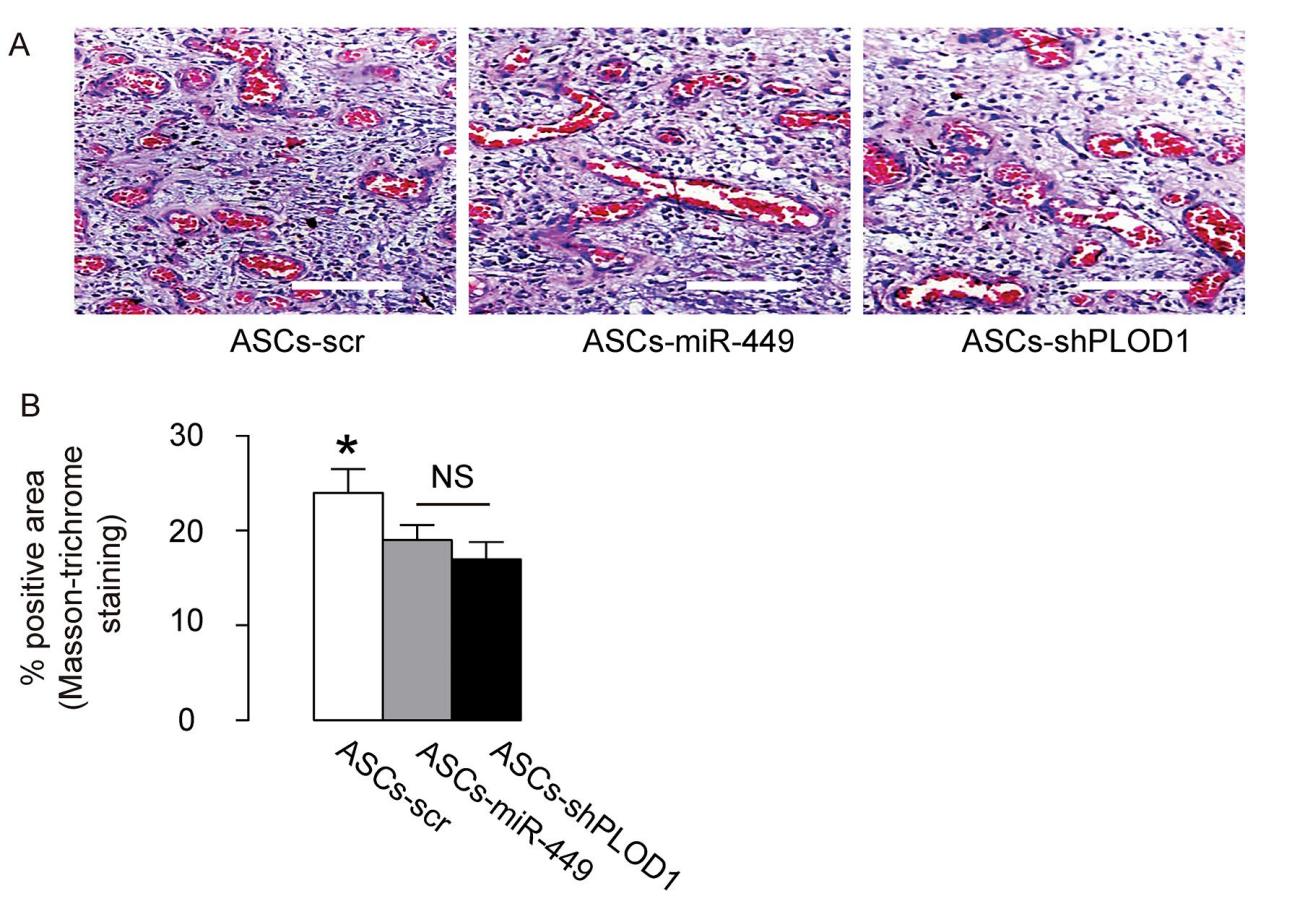
**Transplantation of either ASCs-miR-449 or ASCs-shPLOD1 reduces levels of fibrosis than ASCs during facial nerve regeneration in rats.** (**A-B**) The effects of transplantation of ASCs-miR-449, ASCs-shPLOD1 and control ASCs-scr on the fibrosis were evaluated by Masson-trichrome staining 8 weeks after the surgery in rats, shown by representative images (**A**), and quantification (**B**). *p<0.05. NS: non-significant. N=10. Scale bars are 50µm.

Then we evaluated the effects of transplantation of ASCs-miR-449, ASCs-shPLOD1 and control ASCs-scr on the fibrosis 8 weeks after surgery in rats. We found that transplantation of either ASCs-miR-449 or ASCs-shPLOD1 significantly reduced the fibrosis at the site of injury, compared to transplantation of ASCs-scr, shown by representative images (Figure 5A), and quantification (Figure 5B). These data suggest that transplantation of either ASCs-miR-449 or ASCs-shPLOD1 may improve the anti-fibrotic effects of ASCs.

### Mechanisms underlying the improved anti-fibrotic potential by PLOD1 suppression in ASCs

**Figure 6:**
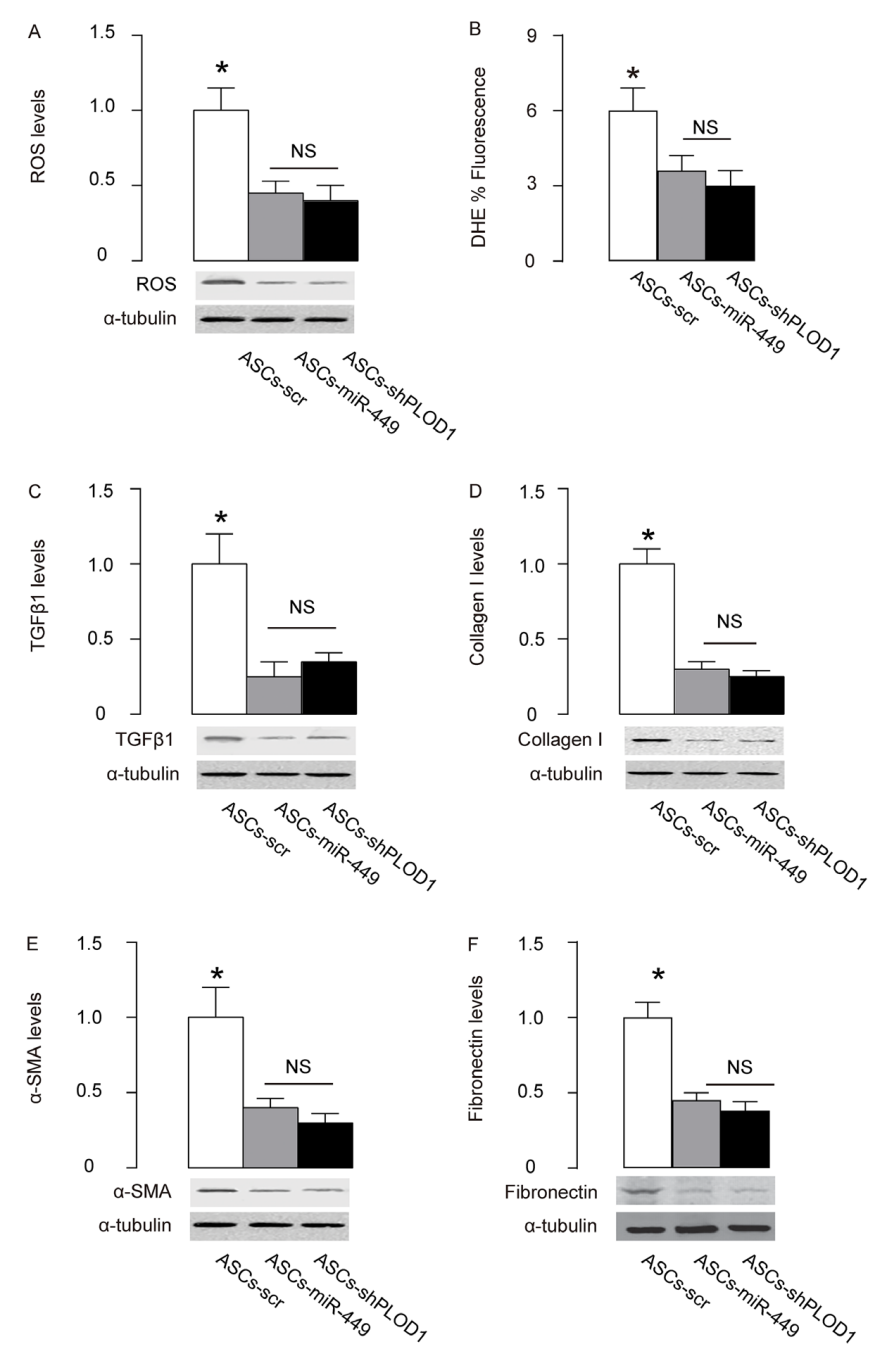
**Mechanisms underlying the improved anti-fibrotic potential by PLOD1 suppression in ASCs.** (**A**) Western blotting for ROS. (**B**) DHE assay. (**C-F**) Western blotting for TGFβ1 (**C**), Collagen I (**D**), α-SMA (**E**) and fibronectin (**F**), at the site of the skin injury. *p<0.05. NS: non-significant. N=10.

Finally, we examined the mechanisms underlying the improved anti-fibrotic potential by PLOD1 suppression in ASCs. Reactive oxygen species (ROS) is critical for fibrogenesis. We examined the levels of ROS at the site of injury and found that transplantation of either ASCs-miR-449 or ASCs-shPLOD1 significantly reduced the ROS levels at the injury site, compared to transplantation of ASCs-scr, by Western blotting (Figure 6A) and by DHE assay (Figure 6B). Transition of myofibroblasts from fibroblasts plays a pivotal role in fibrogenesis. Since α-SMA and TGFβ1 are specific markers for myofibroblasts, we analyzed the levels of these two proteins, as well as a fibrotic marker Collagen I and fibronectin at the site of skin injury. We found that transplantation of either ASCs-miR-449 or ASCs-shPLOD1 significantly reduced the TGFβ1 levels (Figure 6C), Collagen I levels (Figure 6D), α-SMA levels (Figure 6E), and fibronectin levels (Figure 6F) at the site of the injury, compared to transplantation of ASCs-scr. Thus, suppression of PLOD1 levels in ASCs either directly by shPLOD1 or indirectly by miR-449 may substantially improve the anti-fibrotic potential of ASCs during facial nerve regeneration.

## DISCUSSION

The cellular and molecular mechanisms underlying facial nerve regeneration remain poorly understood. Inferior healing affects millions of people worldwide each year. ASCs have the advantage of good abundancy, accessibility and characteristics [6–8]. ASCs share many properties with mesenchymal stem cells (MSCs) but have some advantage. For example, ASCs are positive for CD9, CD29, CD44, CD71, CD73, CD90 and CD105, but negative for CD11b, CD14, CD18, CD31, CD45 and CD56 [24]. The advantages of using ASCs for allogeneic transplantation are their non-invasive obtain, low immunological profile characterized with low expression of HLA-DR class II histocompatibility antigens and high expression of HDLA-ABC class I histocompatibility proteins [25]. Moreover, ASCs express 600 times higher levels of fibroblast-like and alkaline-phosphatase-positive colony-forming units (CFU-F) than bone marrow derived MSCs [26], which renders them to expand faster and longer [27].

It is proposed that ASCs may produce and secrete some trophic factors to enhance tissue regeneration, as well as release some other factors to antagonize the pro-fibrotic factors at the site of wound healing. Moreover, ASCs may adjust inflammation in healing site [9–12].

Fibrosis may impair tissue regeneration, leading to compromised function or dysfunction [22]. Hence, suppression of fibrogenic effects of ASCs may further improve their therapeutic effects on facial nerve regeneration.

A common denominator of fibrosis is the presence of elevated levels of collagen type I, which is deposited by myofibroblasts activated by profibrotic cytokines like TGFβ1. Pyridinoline cross-links are then required to stabilize collagen fibrils, and this process is regulated by PLOD family members including PLOD1, PLOD2 and PLOD3. Collagen containing pyridinoline cross-links are resistant to collagenase-mediated degradation and thereby cause fibrotic lesion. Thus, reduction in pyridinoline cross-links may enable the formation of a non-fibrotic matrix to attenuate scar formation [21]. Here, we detected high PLOD1 in ASCs and thus reduction in PLOD1 levels appeared to be an attractive strategy to interfere with a critical step during fibrosis.

Here, we used two approaches to knockdown PLOD1 in ASCs. First, we used a shRNA to directly knockdown PLOD1. Second, we used a miRNA. MiRNAs are 20~22 nucleotides long non-coding RNAs and are the shortest functional eukaryotic RNAs [28]. Most of miRNAs combine to 3'-UTR of genes by imprecisely binding. After binding, genes are silenced because of alternation of spatial structure [28]. MiRNAs play an important role in various biological processes, such as regulation of cell differentiation, cell identity determination, apoptotic cell death, cell migration and cell cycles, et al [29–32]. Specifically, miR-449 is a miRNA that was found to regulate Notch signaling [33], CDK/Rb/E2F1 [34], and RET kinase/Wnt signaling [35]. However, a role of miR-449 in control of PLOD1 and in regulation of fibrosis and scar formation has not been reported [36–43].

Both strategies achieved satisfactory effects on the anti-fibrotic potential of ASCs. We feel that each strategy should have its advantage and disadvantage. For direct suppression of PLOD1 by shPLOD1, the advantage is that the depletion of PLOD1 is straightforward and unlikely has any off-targeting effects since a scr has been used as a control. However, both PLOD1 mRNA and protein were altered by shPLOD1, and thus it is unknown the effects of reduced PLOD1 mRNA on the cell biology. For indirect suppression of PLOD1 by miR-449, the advantage is that the PLOD1 mRNA is not altered and thus there should be little feedback effects at the mRNA level. Moreover, alteration of a protein level through post-transcriptional control is always an effective and healthy way to modulate cells. However, miR-449 may have target genes other than PLOD1. Thus, the non-specific effects on the ASCs as well as their therapeutic effects on nerve regeneration are still uncertain and required further investigation. In future, further analyses of the non-PLOD1 targets for miR-449 may be helpful for the effects of miR-449-modulation on the therapeutic effects of miR-449. Here we provided evidence to demonstrate a useful approach by altering the levels of PLOD1 in ASCs to improve their application in facial nerve regeneration.

## MATERIALS AND METHODS

### Animals

Male syngeneic Lewis rats aged 16 weeks were purchased from SLAC Laboratory Animal Co. Ltd (Shanghai, China). The rats were maintained at 21°C, under a 12-hour light/dark cycle, and had free access to standard food and water. All experimental procedures were performed in accordance with the guidance for the Care and Use of Laboratory Animals, which was proved by the research committee of Tongji University.

### Preparation and differentiation of ASCs

Adipose tissue was obtained from the dorsocervical subcutaneous region of 16-week-old male syngeneic Lewis rats. After careful rinsing, the adipose tissue was cut into small pieces of 1mm in diameter, and then incubated with 0.2% collagenase I (Sigma-Aldrich, St. Louis, MO, USA) in a rotator at 37^0^C for 45 minutes for digestion. Afterwards, the collagenase I was neutralized with Dulbecco’s modified Eagle’s medium (DMEM; Gibco, San Diego, CA, USA) supplemented with 10% fetal bovine serum (FBS; Gibco) to stop digestion and then the suspension was filtered through a 200 mm nylon mesh to discard the undigested tissue. The obtained cells were re-suspended in DMEM supplemented with 15% FBS. After 10 passages’ selection of attached cells, the cells were sorted for Stro-1 (Becton-Dickinson Biosciences, San Jose, CA, USA) by flow cytometry to get rid of contaminating cells. A positive clone was then subjected to chondrogenetic, osteogenic, and adipogenic differentiation assays for phenotype confirmation, using Osteocyte Differentiation Toolkit [American Type Culture Collection (ATCC), Rockville, MD, USA; Catalog number: PCS-500-052], Adipocyte Differentiation Toolkit (ATCC; Catalog number: PCS-500-050) and Chondrocyte Differentiation Tool (ATCC; Catalog number: PCS-500-051), respectively. Alcian blue staining, Von Kossa staining and Oil red O staining were then performed for detecting differentiated chondrocytes, osteocytes and adipocytes, respectively. Rat fibroblasts (RT2) were purchased from ATCC (Catalog number: CRL-1764). Human skin fibroblasts (HSFs) were purchased from ATCC (Catalog number: SCRC-1041). Recombinant human TGFβ1 (Sigma-Aldrich) was added to cultured cells at a dose of 20ng/ml.

### Transfection and transduction of ASCs

Transfection of ASCs were done using plasmids carrying miR-449, or antisense for miR-449 (as-miR-449), or a scrambled sequence (scr) as a control, with Lipofectamine 3000 reagent (Invitrogen, St. Louis, MO, USA). ASCs were transduced with an adeno-associated virus (AAV) carrying short-hairpin small interfering RNA for PLOD1 (shPLOD1) and a green fluorescent protein (GFP) reporter (connected with a 2A sequence) under a CMV promoter, or an AAV carrying miR-449 and a GFP reporter (connected with a 2A sequence) under a CMV promoter, or a control AAV carrying a GFP reporter and scr. Human embryonic kidney 293 cell line (HEK293, ATCC) was used for virus production. A pAAV-CMV-GFP plasmid (Clontech, Mountain View, CA, USA), a packaging plasmid carrying the serotype 8 rep and cap genes, and a helper plasmid carrying the adenovirus helper functions (Applied Viromics, LLC. Fremont, CA, USA) were used for generation of the viruses. The viruses were purified using CsCl density centrifugation and then titrated by a quantitative densitometric dot-blot assay. The ASCs cells were incubated with AAVs at a multiplicity of infection (MOI) of 150 for 4 hours to transduce the cells. Twelve hours after infection, the transduced cells were purified by flow cytometry base on GFP expression.

### Flow cytometry

For flow cytometric analysis, GFP was detected by direct fluorescence. Data were analyzed using FlowJo software (Flowjo LLC, Ashland, OR, USA).

### Animal model and transplantation of ASCs

Lewis rats were anesthetized with 4% isoﬂurane, after which a preauricular incision with a marginal mandibular extension was made on the left side of the face of the rats to expose the buccal and marginal mandibular branches of the facial nerve and parotid gland. The marginal mandibular branch was transected and ligated with 7-0 nylon sutures (John-son & Johnson, New Brunswick, NJ, USA). A 6-mm defect was created in the buccal branch. For grafting of 10^6^ ASCs, a 30-minute saline flushing on the nerve conduits was performed in advance.

### Functional evaluation of vibrissae movement

Every two weeks after surgery, the left-sided vibrissae movements (whisking) were assessed using a 5-point scale as following: score 0 represents no movement; score 1 represents barely detectable movement; score 2 represents less significant movement; score 3 represents significant but asymmetric movement; score 4 represents symmetric movement. The grades of the observed vibrissae movements were recorded and assessed.

### Electrophysiological assessment of CAMPs

After anesthesia of the rats, two stimulation electrodes were placed subcutaneously just in front of the anterior edge of the parotid gland, one above and one below the left buccal branch of the facial nerve. A monopolar recordings electrode was placed between the middle vibrissal rows, while a reference electrode was placed close to the recorded ipsilateral vibrissal muscles. Additionally, a ground electrode was inserted in the front of the head. The supramaximal compound muscle action potential (CMAP) was recorded by progressive incremental stimulations. The CMAP peak amplitude, duration, and CMAP latency were all assessed.

### Histology

Masson-trichrome staining was performed with a Trichrome Stain (Masson) Kit (Sigma-Aldrich). Myelinated ﬁbers was heat-stained with 0.5% Toluidine Blue (Sigma-Aldrich). The number of myelinated ﬁbers in the middle portion (3.5 mm from the proximal end) of the regenerated facial nerve specimens was counted.

### DHE assays for ROS production

ROS production was detected using dihydroethidium (DHE). Briefly, samples were preincubated with 20 μmol/l DHE for 1 hour, treated with alkylating agents, after which the red fluorescence was monitored at Ex/Em 520/610nm by Fluorometer (Thermo Fisher, Waltham, MA, USA). Delta fluorescence over a 12-hour period was calculated, normalized to cellular DNA content, and then expressed as % fluorescence compared with controls.

### Western blot

Proteins were isolated from cultured cells or rat skin tissue at the injured site. Primary antibodies were rabbit anti-PLOD1, anti-ROS, anti-α-SMA, anti-TGFβ1, anti-collagen I and anti-α-tubulin (Cell Signaling, Carpinteria, CA, USA). Secondary antibody is HRP-conjugated anti-rabbit (Dako, Carpinteria, CA, USA). Figure images were representative from 5 repeats in one group, and NIH ImageJ software (Bethesda, MA, USA) was used for image acquisition and densitometric analysis of the gels.

### Quantitative PCR (RT-qPCR)

Total RNA was extracted from cells using miRNeasy mini kit (Qiagen, Hilden, Germany) for cDNA synthesis and RT-qPCR performed in duplicates with QuantiTect SYBR Green PCR Kit (Qiagen). All primers were purchased from Qiagen. Data were collected and analyzed using 2-△△Ct method. Values of genes were first normalized against α-tubulin, and then compared to experimental controls.

### MicroRNA target prediction and 3'-UTR luciferase-reporter assay

The target miRNAs for PLOD1 was determined by TargetScan, using the context++ score system, as described [23]. The dual-luciferase reporter plasmids, p3’-UTR-PLOD1 (containing the wild-type PLOD1 3’-UTR binding site in luciferase reporter plasmid and p3’-UTR-PLOD1-mut (containing the mutant PLOD1 3’-UTR; mut) were constructed in RiboBio Co. Ltd (Shanghai, China). For the luciferase assay, the constructed 3’-UTR plasmid and miR-449/as-miR-449 were co-transfected into ASCs using LipofectamineTM 3000. The luciferase activity was detected with the dual-luciferase reporter assay system (Promega, Shanghai, China) after co-transfection of the cells for 48 hours, following the manufacturer’s protocol.

### Statistical analysis

GraphPad prism software (GraphPad Software, Inc. La Jolla, CA, USA) was used for statistical analyses. Unpaired two-tailed Student t test was applied for comparison between two groups. One-way ANOVA with a Bonferroni correction was applied for comparison among several groups. Data were represented as mean ± SD and were considered significant if p<0.05.
